# CD83 expression regulates antibody production in response to influenza A virus infection

**DOI:** 10.1186/s12985-020-01465-0

**Published:** 2020-12-10

**Authors:** Madhav Akauliya, Avishekh Gautam, Sony Maharjan, Byoung Kwon Park, Jinsoo Kim, Hyung-Joo Kwon

**Affiliations:** 1 Department of Microbiology, College of Medicine, Hallym University, Chuncheon, 24252 Republic of Korea; 2 Institute of Medical Science, College of Medicine, Hallym University, Chuncheon, 24252 Republic of Korea

**Keywords:** Antibody production, B cells, CD83, Influenza A virus, Peritoneal cavity

## Abstract

**Background:**

CD83 is known to regulate lymphocyte maturation, activation, homeostasis, and antibody response to immunization and infection. While CD83 has a major part in B cell function, its role in influenza A virus infection has not yet been investigated.

**Methods:**

We investigated the role of CD83 using C57BL/6J wild type mice and CD83 knockout (KO) mice after intraperitoneal administration of the influenza A/WSN/1933 virus. We analyzed cells of the peritoneal cavity, splenocytes, and cells of the bone marrow with FACS to investigate CD83 expression and cell population change in response to the virus infection. ELISA was performed with sera and peritoneal cavity fluids to detect A/WSN/1933 virus-specific IgG and the subclasses of IgG.

**Results:**

FACS analysis data showed a transient but distinct induction of CD83 expression in the peritoneal B cells of wild type mice. CD83 KO mice exhibited a delayed recovery of B cells in the bone marrow after influenza virus infection and overall, a smaller T cell population compared to wild type mice. The peritoneal cavity and serum of the wild type mice contained a high titer of IgG within 14 days after infection, whereas the CD83 KO mice had a very low titer of IgG.

**Conclusions:**

These results show the importance of CD83 in lymphocytes homeostasis and antibody production during influenza A virus infection.

## Background

CD83, an evolutionary conserved member of immunoglobulin, is a highly glycosylated type 1 transmembrane glycoprotein composed of 175 amino acids in mice [1] and 186 amino acids in humans [2]. CD83 consists of a variable extracellular Ig-like domain, transmembrane (TM) domain, and intracellular C-terminal cytoplasmic domain sharing 63% homology of amino acids between mice and humans [3]. CD83 is expressed in mature dendritic cells (DCs) and is an activation marker for DCs [3]. However, CD83 is also expressed in natural killer cells [4], activated macrophages [5, 6], neutrophils [7, 8], and activated T cells and B cells [2, 9–11]. CD83 is also expressed on thymic epithelial cells, where it contributes to the selective development and maturation of CD4^+^ T cells [12].

The TM domain of CD83 promotes and stabilizes the cell surface expression of major histocompatibility complex II (MHC II) and CD86 on DCs by inhibiting their association with membrane-bound E3 ubiquitin ligase MARCH1 thereby affecting the activation of CD4^+^ T cells and B cells [13]. CD83 is required for the differentiation and stability of regulatory T (Treg) cells [14]. Moreover, CD83 also promotes the expansion and antigenic specificity of CD8^+^ T cells [15].

Immature B cells beyond the pre-B cell stage, after acquiring functional B cell receptors (BCRs), express a low level of CD83, which has an important role in B cell activation, maturation, homeostasis, effective functioning, longevity, and germinal center response [16–18]. CD83 also reduces the sensitivity of BCRs preventing the overstimulation of B cell [19]. Furthermore, the CD83 antibody in the human system was shown to make B cells unresponsive to antigens, along with a reduced response to CD4^+^ T cells [20].

Because CD83 regulates the development and function of various immune cells, it also modulates the immune response during infections. Infection by different viruses leads to the degradation of membrane CD83 in dendritic cells, interfering with the maturation and immune response of DCs [21–24]. For example, upon hepatitis B virus infection in humans, DCs exhibit a reduced capacity for antigen presentation, cytokine production, phagocytosis, and migration [22]. Previous studies have shown that CD83 also affects B cell function during infection. Mice constitutively expressing CD83 under MHC I promoter (CD83Tg) showed reduced production of antigen-specific antibody production upon infection with *Leishmania major* and *Trypanosoma cruzi* [11]. Similarly, in vitro stimulation of CD83Tg B cells with LPS resulted in enhanced interleukin-10 (IL-10) production along with diminished Ig secretion [25].

Although CD83 is important for B cell function, its role in influenza A virus infection has not yet been investigated. Previously, we showed that intraperitoneal infection with the influenza A/WSN/1933 virus caused a transient but substantial depletion of B cells in the peritoneal cavity [26]. Because B cell function is important to combat virus infection, we investigated the modulation of the B cell and T cell population at different time points after influenza A virus infection and confirmed the requirement of CD83 in the virus-specific antibody production using CD83 knockout (KO) mice.

## Methods

### Cell line and viruses

Madin–Darby Canine Kidney (MDCK) cell lines used for this study were purchased from the American Type Culture Collection (ATCC, Manassas, VA, USA) and grown on Minimum Essential Medium (MEM) with 10% fetal bovine serum (FBS), 100 µg/ml streptomycin and 100 U/ml penicillin. The influenza A virus, A/WSN/1933 (H1N1), was obtained from Professor Man-Seong Park (Korea University, Seoul, Korea). The virus was amplified using specific-pathogen-free (SPF) embryonated eggs followed by infecting the MDCK cell lines. MDCK cells (2 × 10^5^/well) were cultured in 6-well plates using MEM media containing 10% FBS at 37 °C overnight in a CO_2_ incubator. After the overnight culture, the cells were washed with PBS, and each well was infected with the influenza A virus at MOI 0.01 in MEM media containing 1 µg/ml l-tosylamide-2-phenylethyl chloromethyl ketone (TPCK)-treated trypsin and then incubated at 37 °C in CO_2_ incubator. After 1 h incubation, the supernatants were removed and then cultured for 3 days in MEM media containing 0.3% BSA at 37 °C in a CO_2_ incubator. The virus culture supernatants were collected and centrifuged at 2,000 rpm for 10 min at 4 °C to remove the cell debris. The quantification of the amplified viruses was performed by plaque assay. MDCK cells (6 × 10^5^/well) were cultured in 6-well plates. The cells were cultured, washed with PBS, and infected with the amplified influenza A virus culture supernatants after a ten-fold serial dilution. After 1 h incubation, the supernatants were removed and overlaid with 2 ml of DMEM/F12 media containing 2 mM glutamine, 4% BSA, 10 mM HEPES, 2.5% sodium bicarbonate, 50 mg/ml DEAE dextran, 1 µg/ml l-tosylamide-2-phenylethyl chloromethyl ketone (TPCK)-treated trypsin, 100 U/ml penicillin, 100 μg/ml streptomycin and 0.6% immunodiffusion-grade agar. After 72 h incubation, the plates were stained with crystal violet (0.1% crystal violet in 20% methanol) for 1 h. The plaques were counted to determine the virus titers. The entire process for the virus preparation and cell culture procedure was conducted in a biosafety level 2 laboratory.

### Mice

Eight-week-old female C57BL/6J mice were purchased from NARA Biotech, Inc. (Seoul, Korea), and C57BL/6J CD83 KO mice (B6.129S4-*Cd83*^*tm1Tft*^/J, JAX stock #017703) were purchased from The Jackson Laboratory (Bar Harbor, ME, USA). The mice were maintained at the Animal Center of Hallym University under specific pathogen-free conditions (20–25 °C, 40–45% humidity, 12 h light/dark cycle, and food and water access, ad libitum). Mice experiments were performed in a biosafety level 2 facility in the Hallym Clinical and Translational Science Institute.

### Virus infection

A/WSN/1933 virus inactivation was performed by 254 nm UV light exposure with 1,500 mW/s/cm^2^ UV for 15 min from a height of 5 cm. The virus inactivation was verified by a plaque assay with MDCK cells [27]. The live A/WSN/1933 virus or UV-inactivated A/WSN/1933 virus was intraperitoneally infected at a dose of 5 × 10^6^ pfu per mouse and CD83 expression in the peritoneal cavity, bone marrow and spleen cells was observed at 1 h, 3 h, 6 h, and 24 h after infection. Changes in lymphocyte population were observed at 5, 7 and 14 days after live A/WSN/1933 virus infection. The production of virus-specific antibody was measured at 14 days after live A/WSN/1933 virus infection.

### FACS analysis

C57BL/6J wild type or C57BL/6J CD83 KO mice were infected intraperitoneally with 5 × 10^6^ pfu of A/WSN/1933 virus. After the virus infection, cells of the peritoneal cavity, splenocytes, and cells of the bone marrow were harvested in RPMI 1640 media containing 5% FBS. The cells were centrifuged at 1,200 rpm and 4 °C for 5 min, and the pellets were treated with RBC lysis buffer (20 mM Tris HCl and 140 mM NH_4_Cl) for 5 min. After washing with RPMI media, total cell numbers of peritoneal cavity, spleen and bone marrow were counted and analyses of cell populations were performed as described previously (26). The cells were washed with FACS buffer (1% FBS in PBS) and transferred into Falcon tubes and then treated with 10 µg/ml anti-FcγRII/III antibody (Catalogue No: 553142, BD Biosciences, San Jose, CA, USA). To analyze lymphoid population, PerCP Cy5.5-conjugated anti-CD3 antibody (Catalog No: 551163, BD Biosciences), Pacific blue ef450-conjugated anti-CD4 antibody (Catalog No: 558107, BD Biosciences), FITC-conjugated anti-CD8 antibody (Catalog No: 553031, BD Biosciences), APC-Cy7-conjugated anti-CD19 antibody (Catalog No: 557655, BD Biosciences), PE-conjugated anti-CD23 antibody (Catalog No: 12-0232-81, eBioscience, San Diego, CA, USA), and APC-conjugated anti-CD83 antibody (Catalog No: 558208, BD Biosciences) were used. We pre-gated peritoneal cells, bone marrow cells, splenocytes (FSC^low^SSC^low^) for the enriched lymphoid population and then analyzed specific cell population with a FACSCanto™ II Flow Cytometry (BD Biosciences).

### ELISA

For the detection of A/WSN/1933 virus-specific IgG, the subclasses of IgG, ninety-six well Immuno plates (Nunc™, Roskilde, Denmark) were coated with the A/WSN/1933 virus. After overnight incubation at 4 °C, the plates were washed three times with PBST (0.1% Tween-20 in PBS) and blocked with 1% BSA for 1 h and then loaded with three-fold dilutions of sera and peritoneal supernatants in PBST. After incubation at room temperature for 2 h, the plates were washed three times with PBST and goat anti-mouse IgG/IgG1/IgG2a/IgG2b/IgG3 antibodies labeled with Horseradish peroxidase (Southern Biotechnology Associates, Inc. Birmingham, AL, USA) with dilutions 1:500 were used to bind total IgG and each subclasses antibody. The plates were incubated for 1 h at room temperature, and then, TMB substrate solution was added (Kirkegaard and Perry Laboratories, Gaithersburg, MD, USA). The absorbance was measured by a Spectra Max 250 microplate reader (Molecular Devices, Sunnyvale, CA, USA) at 450 nm.

### Statistical analysis

Results are shown as the mean ± standard deviation. The statistical significance of the differences between the two samples was evaluated using Student’s t-test with P < 0.05 as the threshold for statistical significance.

### Ethical approval

Mice experimental procedures were undertaken following the guidelines of Laboratory Animals of the National Veterinary Research and Quarantine Service of Korea. Animal use and related experimental procedures were approved by the Institutional Animal Care and Use Committee of Hallym University (Permit Number: Hallym R2/2018-25). Mice were anesthetized with 3% to 5% isoflurane (Pharmaceutical, Seoul, Korea) inhalation to minimize any pain. The mice were sacrificed by CO_2_ inhalation after the experiments and euthanized by CO_2_ inhalation if they lost 25% of their baseline adult body weight or if they revealed evidence of debilitation, pain or distress such as a hunched posture, rough hair coat, reduced food consumption, emaciation, inactivity, ambulation difficulty, and respiratory problems, and all efforts were made to limit suffering.

## Results

### CD83 expression in peritoneal B cells upon influenza A virus infection

**Fig. 1:**
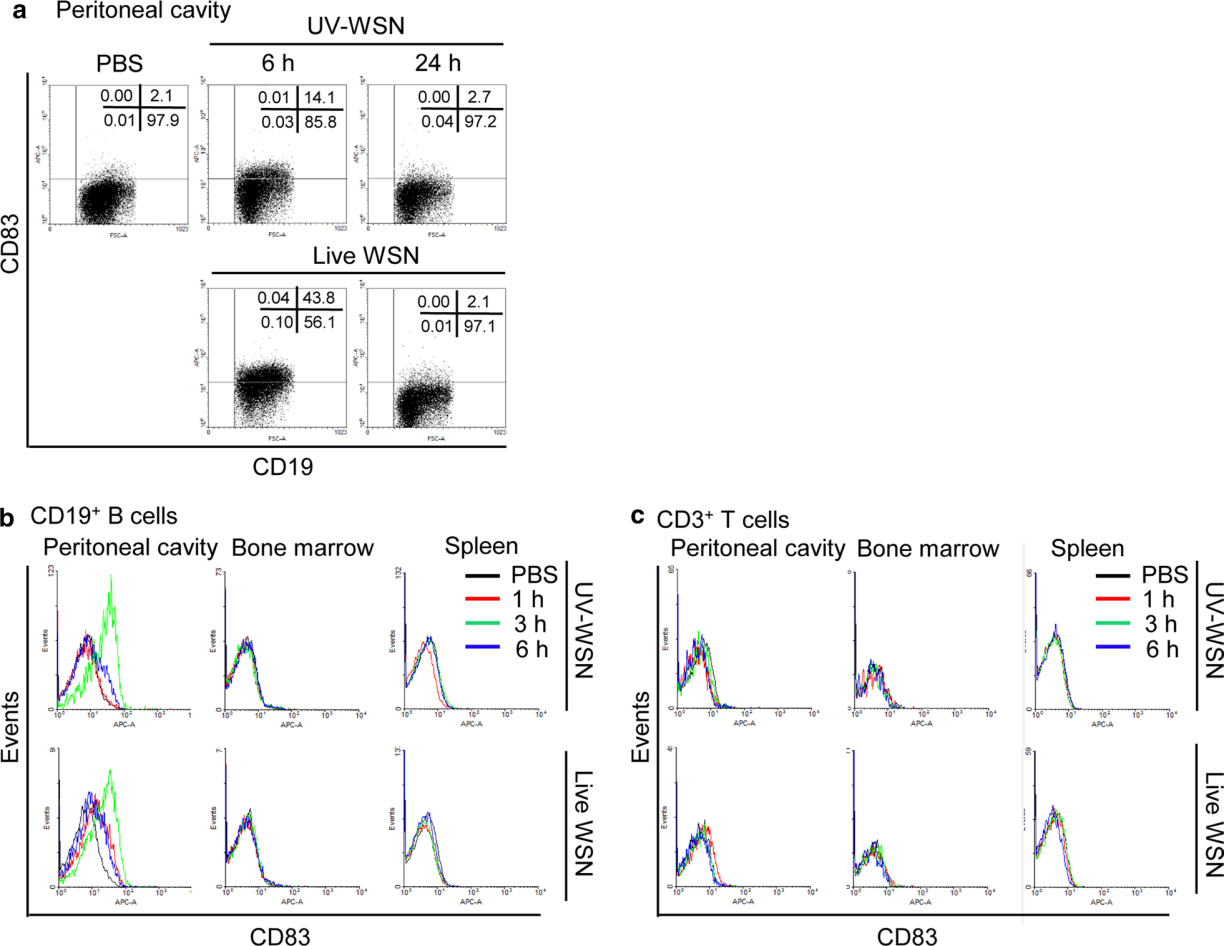
CD83 expression in peritoneal cells, bone marrow cells, and splenocytes after intraperitoneal injection of A/WSN/1933 virus. Cells were collected at 1, 3, 6, and 24 h after intraperitoneal injection of live A/WSN/1933 virus (WSN) or UV-inactivated A/WSN/1933 virus (UV-WSN) in C57BL/6J mice, and CD83 expression was analyzed by FACS. **a** CD83 expression in CD19^+^ B cells of the peritoneal cavity at 6 and 24 h after infection. **b**, **c** CD83 expression in CD19^+^ B cells (**b**) and CD3^+^ T cells (**c**) of the peritoneal cavity cells, bone marrow cells, and splenocytes at 1, 3 and 6 h after infection

B cells perform a vital function in combating infection through the production of antibodies and cytokines and antigen presentation to CD4^+^ and CD8^+^ T cells [28]. Because CD83 has an important role in the development and function of B cells and T cells [17], we investigated the role of CD83 in B cell function during influenza A virus infection in mice. To determine the effect of influenza A virus on CD83 expression in peritoneal cells, C57BL/6J wild type mice were intraperitoneally injected with 5 × 10^6^ plaque forming units (pfu)/mouse of live or UV-inactivated A/WSN/1933 virus. FACS analysis results showed that the expression of CD83 on CD19^+^ peritoneal B cells was drastically increased at 3 h and then restored to basal level at 24 h after infection with live A/WSN/1933 virus (Fig. 1b). The expression of CD83 on CD19^+^ B cells was also increased at 3 h in the mice infected with UV-inactivated WSN virus; however, the levels of CD83 expression at 6 h were substantially lower than those of live virus-infected mice (Fig. 1a). CD83 expression on CD19^+^ B cells in mice infected with UV-inactivated virus was also restored to basal level at 24 h. The expression of CD83 on CD19^+^ B cells from bone marrow and the spleen was not affected by live or UV-inactivated A/WSN/1933 virus-infection (Fig. 1b). However, CD3^+^ T cells from the peritoneum cavity, bone marrow, and spleen showed no difference in expression of CD83 upon infection with either live or UV-inactivated virus (Fig. 1c). These results show that infection of influenza A virus leads to a transient but distinct increase of CD83 expression in the surface of peritoneal B cells. As overall CD83 expression patterns induced by live or UV-inactivated virus are similar, we then performed further experiments with live A/WSN/1933 virus using PBS as a control.

### CD83 is required for the restoration of lymphocyte populations in the bone marrow after intraperitoneal challenge with influenza A virus

**Fig. 2:**
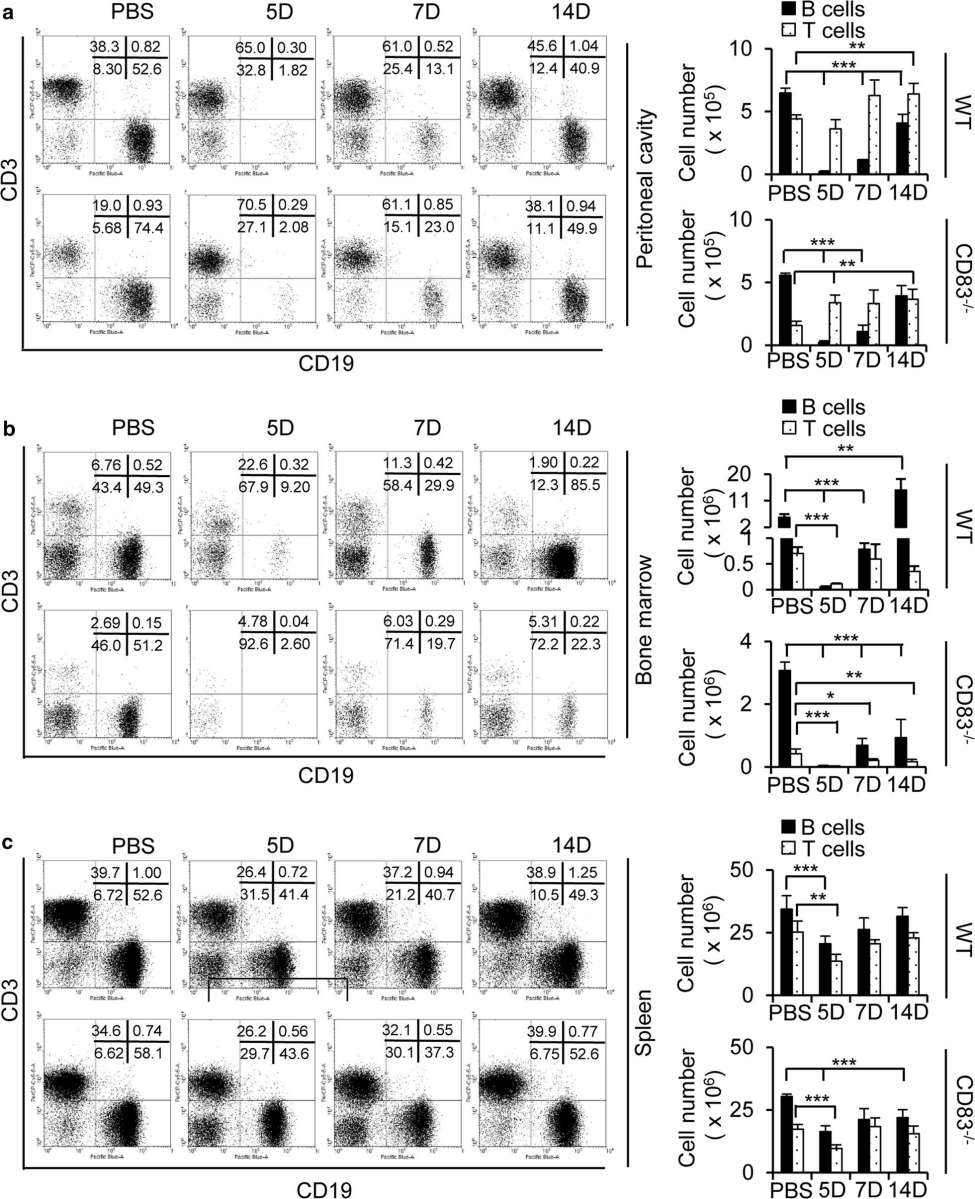
Population of lymphoid cells in the peritoneal cavity fluid, bone marrow, and spleen after intraperitoneal injection of A/WSN/1933 virus in C57BL/6J wild type mice and CD83 KO mice. **a**–**c** Wild type mice and CD83 KO mice (n = 5/group) were injected intraperitoneally with 5 × 10^6^ pfu of A/WSN/1933 virus. Peritoneal cells, bone marrow cells, and splenocytes were harvested at 5, 7, and 14 days after infection. Cells were enumerated and stained with fluorescence-conjugated antibodies to be analyzed by FACS. FSC^low^SSC^low^ cells of peritoneal cavity fluid (**a**), bone marrow (**b**), and spleen (**c**) were sorted into CD19^+^ B cells and CD3^+^ T cells for all experimental groups of mice. The left panel displays the FACS profile with the percentage of the subpopulation in each quadrant. The right panel displays the absolute number of CD19^+^ B cells and CD3^+^ T cells in each subgroup. *P < 0.05, **P < 0.01, ***P < 0.001

Previously, we have reported that intraperitoneal inoculation with the A/WSN/1933 virus induced a substantial but transient B cell depletion at 12 h post-infection, which was partially recovered at 7 days with a gradual restoration to normal levels by 14 days [26]. To investigate the effect of CD83 expression in the lymphocyte population and its restoration pattern in the peritoneal fluid, bone marrow, and spleen, wild type mice and CD83 KO mice were intraperitoneally injected with the live A/WSN/1933 virus at a dose of 5 × 10^6^ pfu/mouse. Peritoneal cells, bone marrow cells, and splenocytes were harvested at 5, 7, and 14 days post-infection, and cell populations were analyzed by flow cytometry. In the wild type mice, depletion of CD19^+^ B cells was observed at 5 days post-infection in the peritoneal cavity (Fig. 2a) and bone marrow (Fig. 2b), followed by a partial recovery at 7 days and restoration to near-normal levels at 14 days post-infection as previously described [26]. In CD83 KO mice, transient depletion and then recovery to the normal level were observed in CD19^+^ B cells of the peritoneal cavity similar to wild type mice (Fig. 2a). However, the CD19^+^ B cells in bone marrow exhibited an incomplete recovery in the CD83 KO mice until 14 days post-infection, suggesting a delay in the restoration process (Fig. 2b). On the other hand, the peritoneal cells exhibited an increment in CD3^+^ T cells for both the wild type and CD83 KO mice after infection although the amounts of T cells at 7 and 15 days post-infection were higher in the wild type mice than in the CD83 KO mice (Fig. 2a). Furthermore, the basal levels of CD3^+^ T cells in the peritoneal cavity of the CD83 KO mice were much lower (about 50%) compared to those of wild type mice (Fig. 2a). The T cell numbers of the bone marrow declined at day 5 and slowly recovered at day 7 and 14 in the wild type mice and CD83 KO mice (Fig. 2b). In the spleen, the CD19^+^ B cells and CD3^+^ T cells were decreased at 5 days post-infection, followed by a partial recovery at 7 days and restoration to near-normal levels at 14 days post-infection with no prominent difference between the wild type mice and CD83 KO mice (Fig. 2c). Collectively, these results show the requirement of CD83 for the homeostasis of T cells in the peritoneal cavity fluids and for the restoration of B cells in the bone marrow following influenza A virus infection.

### Influenza A virus triggers an altered behavior by the T cell subpopulation in the peritoneal cavity of CD83 knockout mice.

**Fig. 3:**
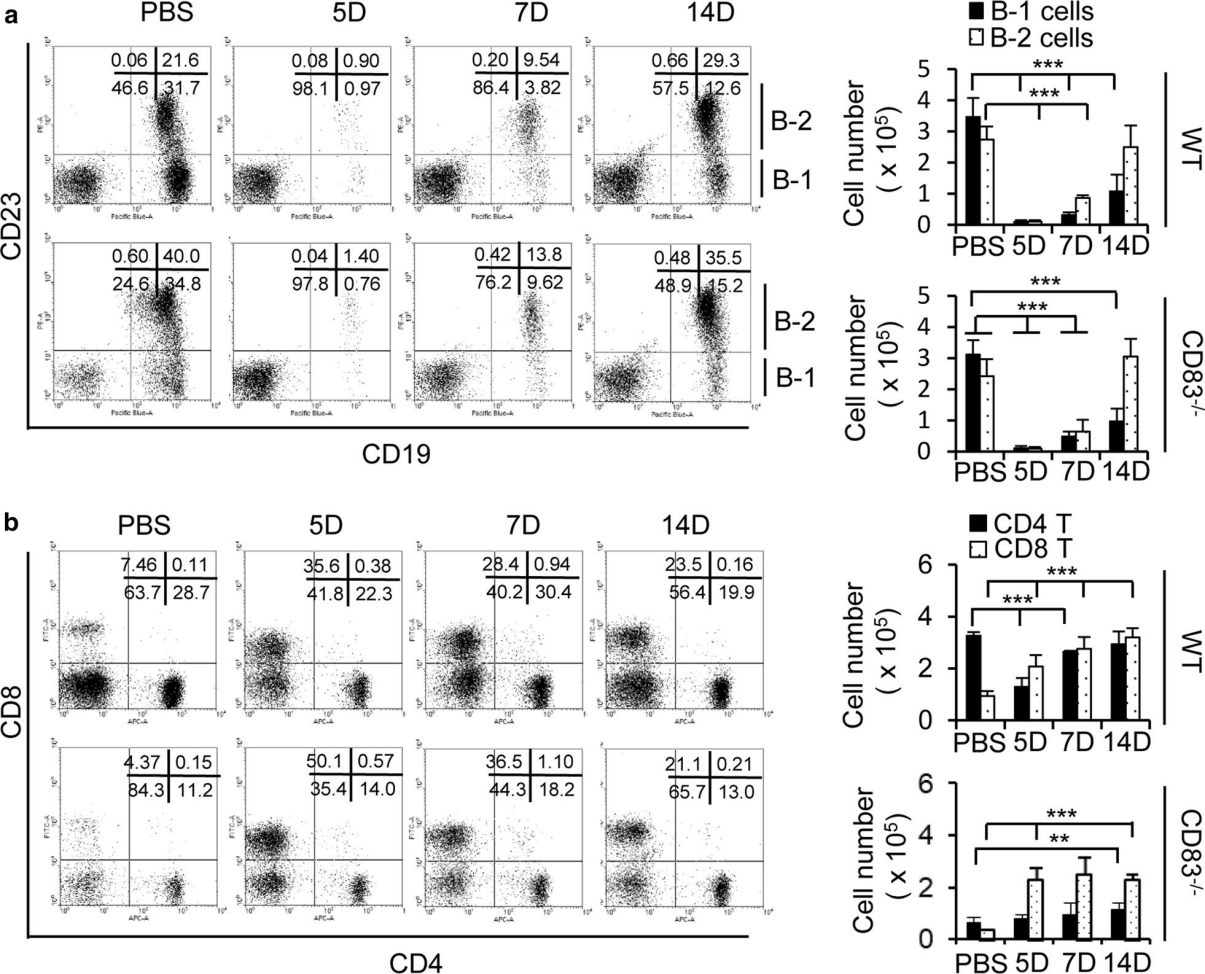
Analysis of B cells and T cells in the peritoneal cavity fluid after intraperitoneal injection of A/WSN/1933 virus in C57BL/6J wild type mice and CD83 KO mice. Wild type mice and CD83 KO mice (n = 5/group) were injected intraperitoneally with 5 × 10^6^ pfu of A/WSN/1933 virus. Peritoneal cells were harvested at 5, 7, and 14 days after infection. **a** FSC^low^SSC^low^ cells of peritoneal cavity fluid were sorted into B-1 cells (CD19^+^, CD23^−^) and B-2 cells (CD19^+^, CD23^+^). **b** FSC^low^SSC^low^ cells of peritoneal cavity fluid were sorted into CD4^+^ and CD8^+^ subsets. The bar diagram on the right side showed the absolute number of cells in each subgroup. **P < 0.01, ***P < 0.001

B cell populations are composed of subclasses B-1 and B-2 cells. B-1 cells, unlike the classical B-2 cells, are characterized by a low level of CD23 expression and participates in the antibody response during vaccination and infection [29]. Therefore, we analyzed the B-1 and B-2 cells in the peritoneal cavity fluids after intraperitoneal infection of live A/WSN/1933 virus. The depletion of both the B-1 and B-2 cells was observed at 5 days post-infection, followed by partial recovery at 7 days in both wild type mice and CD83 KO mice (Fig. 3a). While B-2 cells had a full restoration of their cell numbers at day 14, B-1 cells were unable to recover to a normal level. However, there was no significant difference between the wild type and CD83 KO mice (Fig. 3a). Next, we checked the T cell populations and found that after the virus inoculation, the CD8^+^ T cell number underwent continuous expansion as observed at day 5, 7, and 14 in both the wild type (threefold) and CD83 KO (fivefold) mice (Fig. 3b). In contrast, CD4^+^ T cells exhibited an altered behavior between the wild type and CD83 KO mice. As observed earlier, the T cell population was smaller in the CD83 KO mice [30]. In the wild type mice, the CD4^+^ T cell number was reduced at day 5 post-infection and later was restored to the normal level by day 14. However, the CD4^+^ T cell number mildly increased after infection in the CD83 KO mice; however, the total number of cells in the CD83 KO mice was still much lower compared to the wild type mice (Fig. 3b). Overall, these results suggest that CD83 loss could lead to differential properties, especially in the T cell population in the peritoneal cavity.

### Effect of CD83 expression on antibody production after intraperitoneal challenge with influenza A virus

**Fig. 4:**
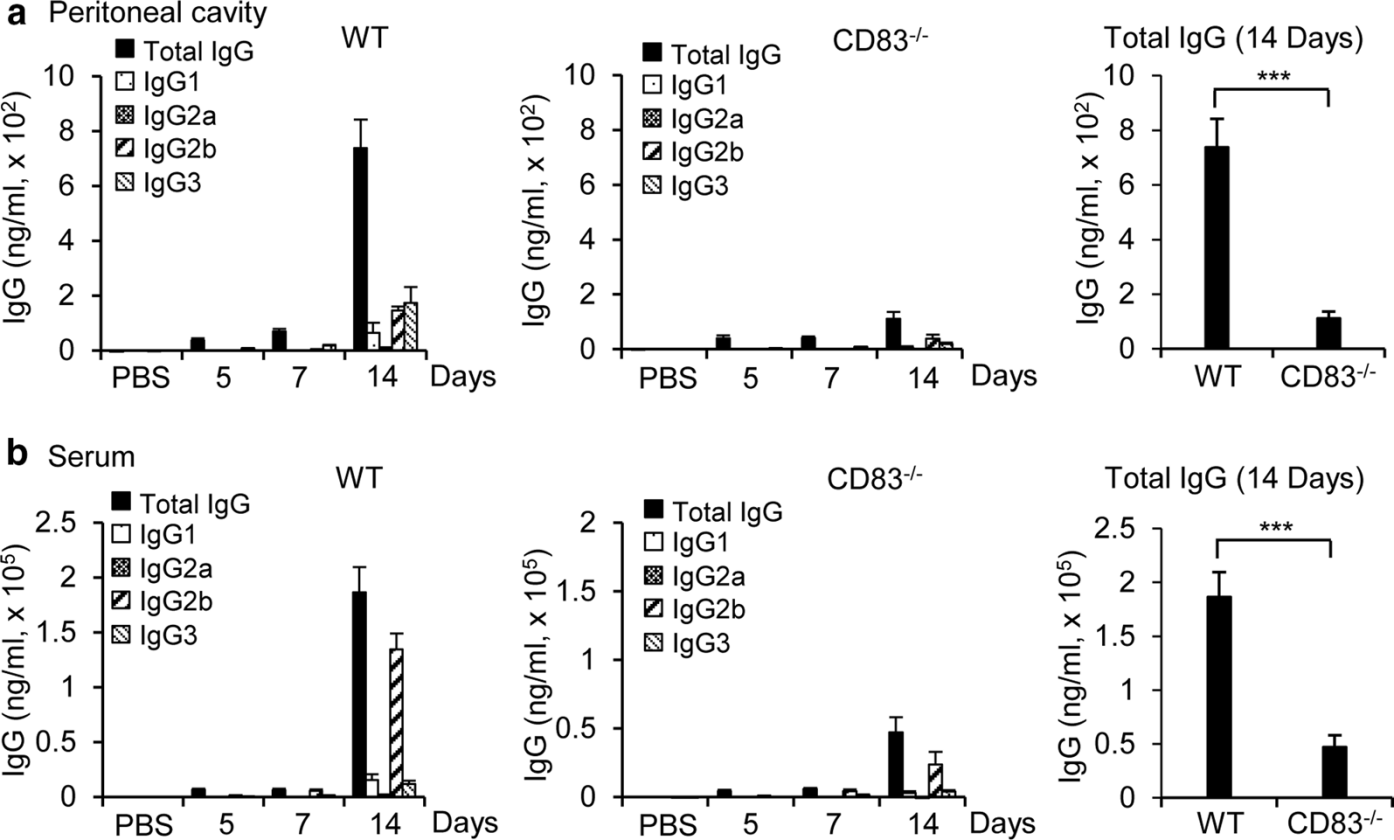
A/WSN/1933 virus-specific antibody production by C57BL/6J wild type mice and CD83 KO mice after intraperitoneal injection of A/WSN/1933 virus. Peritoneal cavity fluids and sera were collected at 5, 7, and 14 days after intraperitoneal injection of live A/WSN/1933 virus in mice (n = 5/group) with a dose of 5 × 10^6^ pfu/mice. The amount of total IgG and IgG subclasses were quantified by ELISA. **a** A/WSN/1933 virus-specific antibody production in the peritoneal cavity fluid. **b** A/WSN/1933 virus-specific antibody production in sera. ***P < 0.001

Upon infection, some of the naïve B cells are converted to plasma cells that produce antibodies against the antigenic components of invading pathogens, which has an essential role in the clearance of pathogens [31]. Antibody production by B cells could be CD4^+^ T cell-dependent or independent [32]. Because the loss of CD83 affected the B cell population and CD4^+^ T cell population after infection, we investigated the effect of CD83 expression on antibody production in response to virus infection by measuring the level of virus-specific IgG and its subclasses in the peritoneal cavity fluids and serum at 5, 7 and 14 days post-infection. There were no detectable virus-specific IgG in the PBS control mice. A negligible amount of the virus-specific IgG was produced in the peritoneal cavity fluids and serum at day 5 and 7 post-infection, which later increased to a considerable value by day 14 in the wild type mice. Notably, IgG2b and IgG3 were the major IgG isotypes in the peritoneal cavity fluids (Fig. 4a), whereas IgG2b was the most prominent in the serum (Fig. 4b). Conversely, the CD83 KO mice failed to produce virus-specific antibodies comparable to the wild type mice (Fig. 4). Although IgG levels were increased at day 14 in the CD83 KO mice, the amount was significantly lower compared to the wild type mice both in the peritoneal cavity fluids (Fig. 4a) and serum (Fig. 4b). Taken together, these results suggest that CD83 has an important role in B cell response to influenza A virus infection with a direct influence on the production of virus-specific antibodies.

## Discussion

Although CD83 is a key component of the immune system that can modulate T cell and B cell function, no studies involving CD83 and influenza virus have been carried out so far. Recently, we demonstrated that intraperitoneal injection of influenza A/WSN/1933 virus induced an efficient immune response and robust virus-specific antibody production by B cells that sufficiently provides cross-protection against other strains of influenza A virus [26]. Here, we investigated the effect of CD83 on B and T cells during influenza virus infection using C57BL/6J wild type mice in parallel with CD83 KO mice. We found that CD83 expression is required for the optimal production of virus-specific antibodies during influenza virus infection.

B cells have a crucial role in combating virus infection via antigen presentation to the T cells and the production of high-affinity antibodies against virus antigens [33]. To explore the function of CD83 in B cells, we examined the expression of CD83 on the cell surface of B cells after intraperitoneal administration of influenza A virus on wild type mice. Influenza A virus infection induced the cell surface expression of CD83 on peritoneal B cells, reaching a plateau within 3 h, without affecting the B cells in the spleen and bone marrow. The expression of CD83 returned to the basal level within 24 h. Although CD83 also has an important role in the development and functioning of T cells [12, 15], influenza A infection did not significantly affect the CD83 expression level on the T cells in our experimental settings. These initial results suggest that CD83 mainly functions through B cells during the early stages of influenza A infection like priming or activation of the B cells.

Previously, we demonstrated that intraperitoneal administration of influenza A virus triggered an increase of T cells and a transient loss of B cells and macrophages, which recovered after 2 weeks post-infection in the peritoneal cavity [26]. Through experiments with CD83 KO mice in this study, it was found that without CD83, the B cell population was recovered in the peritoneal cavity fluids and spleen similarly to wild type, but the B cell recovery in the bone marrow was prominently delayed. Bone marrows possess developing and immature B cells. B cell development within bone marrow and migration into peripheral blood circulation are regulated by expression of cytokines, chemokines and chemokine receptors [34]. For example, up-regulation of CXCR5 in preB cells induced migration to CXCL12 producing cells in bone marrow [35]. The bone marrow specific loss of B cell restoration in influenza A virus-infected CD83 KO mice might have resulted from differential expression of some chemokine receptors on the B cells or altered production of cytokines and chemokines within the various immune compartments. However, further studies are required to clarify this issue.

Importantly, the T cell population was smaller in the peritoneal cavity, bone marrow, and spleen in the CD83KO mice with the highest difference in the peritoneal cavity. B cells can activate T cells via antigen presentation on the MHC molecules, and the activated helper T cells can activate the B cells to enter the germinal center reaction where the activated B cells expand exponentially and later differentiate to memory cells or plasma cells [36, 37]. CD83 is known to stabilize the MHC II molecule on the cell surface by inhibiting its interaction with ubiquitin ligase. Thus, it is plausible that in the absence of CD83, the expansion of CD4^+^ T cells in the peritoneal cavity is impaired, resulting in the delayed activation of B cells and lesser IgG production after influenza A virus infection. Antibodies that are derived from B cells in response to influenza virus are crucial in preventing mortality and morbidity, providing a full recovery, and producing memory cells for future immunity [31]. In this study, mice infected with influenza A virus produced a high amount of IgG after 14 days as measured in the peritoneal cavity fluids and serum. Concomitantly, with a reduced population of CD4^+^ T cells, CD83 KO mice had very low levels of virus-specific antibodies. Previous studies showed that the surface expression of CD83 on B cells is indirectly proportional to the amount of IgG production. Kretschmer et al. showed that CD83Tg overexpressing B cells produce less Igs, whereas CD83 deficient B cells produced higher levels after T-cell dependent and independent immunization [25]. In our experimental conditions, the virus infection induced CD83 expression only transiently, and the expression level returned to the basal level within 24 h post-infection. Therefore, CD83 is likely to affect the activation and proliferation of B cells. The lower number of the CD4^+^ T cell population in the CD83 KO mice [12] could also have affected the antibody production efficacy after virus infection. Further studies are required to dissect the precise role of CD83 on B cells and the interaction of CD83^+^ B cells and CD4^+^ T cells in antibody production. Moreover, the role of CD83 on macrophages and dendritic cells against influenza virus infection needs to be determined in future studies.

Gordon et al. [38] showed that IgG2 deficiency was associated with an increased risk of severe H1N1 infection in obese, pregnant, and immunosuppressed cases leading to the severe pandemic 2009 influenza A infection. Another study reported the effect of age on IgG subtypes after influenza vaccination and found that IgG1 and much more IgG3 were elevated in the serum. The higher level of IgG3 production was found to be positively correlated with the level of tumor necrosis factor-α (TNF-α) and interleukin-6 (IL-6) [39] because IL-6 is the major cytokine produced during influenza infection [40]. In response to the intraperitoneal infection of the influenza A/WSN/1933 virus in C57BL/6J wild type mice, IgG2b and IgG3 were the dominant IgG in the peritoneal cavity fluids whereas IgG2b was the major IgG in the serum. In the CD83 KO mice, both in the peritoneal cavity fluids and serum, IgG was found to be decreased in comparison to the wild type mice. In contrast to our study, previous reports showed that IgG1 was the major subclass in the serum of C57BL/6J mice, while IgG2a was the major one in BALB/c mice infected with the A/Queensland/6/72 (H3N2) influenza virus [41]. Another study showed serum IgG2a as prominent in CBA/CaH mice during intraperitoneal inoculation of A/Queensland/6/72 (H3N2) [42]. Altogether, it is likely that the prevalence of IgG subclass depends on various factors like the type of virus strain, route of inoculation, days of measurement, and types of experimental mice undertaken for the study.

Gender issue related with mouse models of influenza infection is a concerning factor these days as it can interfere experimental outcomes. Epidemiological reports showed that influenza infection causes higher mortality and morbidity in women, especially pregnant women, than in men [43–45]. Previously, we reported that high-dose intraperitoneal infection of influenza A viruses in female mice induced severe sclerosis in the pancreas, disruption of ovarian follicles, and massive infiltration of immune cells in the uterus [46]. On an extension of the study, we investigated implication of CD83 using female mice in this study. Vaccination efficacy against influenza A virus (A/PR8/H1N1) showed higher survival rate, mild clinical disease, and less weight loss in female mice than in male mice [47]. Induction of antibody-mediated immunity and effect of cross-protection by influenza A virus vaccination were higher for females mice than males [48, 49]. Therefore, further studies are required to investigate the effect of CD83 expression on influenza A virus-specific antibody production in male mice and compare the results with those obtained in this study.

## Conclusions

This work showed the important role of CD83 in B and T cell homeostasis and antibody production during influenza A virus infection. These results could provide important insights into future studies that are directed towards antibody response and vaccine production for influenza viruses.

## Data Availability

Not applicable.

## Abbreviations

DC: dendritic cell
ELISA: enzyme-linked immunosorbent assay
FACS: fluorescence-activated cell sorting
FBS: fetal bovine serum
KO: knockout
MDCK: Madin–Darby Canine Kidney
MEM: minimum essential medium
pfu: plaque forming unit
